# Identification of virulence associated loci in the emerging broad host range plant pathogen *Pseudomonas fuscovaginae*

**DOI:** 10.1186/s12866-014-0274-7

**Published:** 2014-11-14

**Authors:** Hitendra Kumar Patel, Maura Matiuzzo, Iris Bertani, Vincent de Paul Bigirimana, Gavin J Ash, Monica Höfte, Vittorio Venturi

**Affiliations:** International Centre for Genetic Engineering and Biotechnology, Trieste, Italy; Department of Crop Protection, Laboratory of Phytopathology, Ghent University, Coupure, Links 653, 9000 Ghent, Belgium; Graham Centre for Agricultural Innovation, School of Agricultural and Wine Sciences, Charles Sturt University, Wagga Wagga, NSW 2650 Australia

## Abstract

**Background:**

*Pseudomonas fuscovaginae* (*Pfv*) is an emerging plant pathogen of rice and also of other gramineae plants. It causes sheath brown rot disease in rice with symptoms that are characterized by brown lesions on the flag leaf sheath, grain discoloration and sterility. It was first isolated as a high altitude pathogen in Japan and has since been reported in several countries throughout the world. *Pfv* is a broad host range pathogen and very little is known about its virulence mechanisms.

**Results:**

An *in planta* screen of 1000 random independent Tn*5* genomic mutants resulted in the isolation of nine mutants which showed altered virulence. Some of these isolates are mutated for functions which are known to be virulence associated factors in other phytopathogenic bacteria (eg. *pil* gene, phytotoxins and T6SS) and others might represent novel virulence loci.

**Conclusions:**

Being an emerging pathogen worldwide, the broad host range pathogen *Pfv* has not yet been studied for its virulence functions. The roles of the nine loci identified in the *in planta* screen are discussed in relation to pathogenicity of *Pfv*. In summary, this article reports a first study on the virulence of this pathogen involving *in planta* screening studies and suggests the presence of several virulence features with known and novel functions in the *Pseudomonas* group of bacteria.

**Electronic supplementary material:**

The online version of this article (doi:10.1186/s12866-014-0274-7) contains supplementary material, which is available to authorized users.

## Background

*Pseudomonas fuscovaginae* (*Pfv*) is a Gram-negative, fluorescent pseudomonad and a member of Gamma proteobacteria [1,2]. *Pfv* is one of the 18 validly described *Pseudomonas* plant pathogenic species, which are part of the oxidase positive cluster [3,4]. This bacterium was first identified and reported as a pathogen of rice (*Oryza sativa*) in the temperate region of Japan in 1976 [2]. It has now been described in several other regions of the world where rice and other gramineae food crops are cultivated including Burundi [5], Madagascar [6], Mexico [7], the Philippines [8], Nepal [9], Brazil [10], China [11], Iran [12] and more recently in Malaysia [13] and Australia [14]. *Pfv* causes brown sheath rot disease in rice and also in other gramineae food crops including maize (*Zea mays*), sorghum (*Sorghum bicolour*) and wheat (*Triticum aestivum*) [5,7]. Brown sheath rot symptoms on rice plants appear at all growing stages. At the seedling stage symptoms start with yellow to brown discoloration on the lower leaf sheath which later turns into grey-brown to dark-brown and ultimately, the infected seedlings rot and die. In mature rice plants *Pfv* symptoms can be observed on flag leaf sheaths, other leaf sheaths and also on the panicles. Under severe infection conditions, the entire leaf sheath becomes necrotic and dries out. Spikelets of emerging panicles may be discoloured, sterile or symptomless, except for small brown spots [1,15].

A successful infection by a phytopathogenic bacterium is not a single step process and is coordinated by several functions including bacterial adherence, movement, colonization, invasion, and suppression of host immunity. Type IV pili are one of the best characterized adhesins in *Pseudomonas* pathogens and it has been shown to be involved in several functions including adhesion in *P. syringae* pv. *phaseolicola* [16], epiphytic fitness and survival in *P. syringae* pv. *syringae* [17] and *P. syringae* pv. *tomato* [18] and also in surface motility and virulence in *P. syringae* pv. *tabaci* [19,20]. The colonization process in *Pseudomonas* plant pathogens has been associated with exopolysaccharides (EPSs) shown to be involved in biofilm formation, epiphytic fitness and virulence [21,22]. The invasion process of plant pathogens has also been linked with secretion of several cell wall degrading enzymes including pectinolytic enzymes, cellulases and lipase through protein secretion systems [23]. Plant pathogenic bacteria also have strategies to suppress the host defense responses induced during the infection process by secretion of effector proteins directly into the host cell through a type III secretion system (T3SS) [24]. In addition to these functions, phytopathogenic pseudomonads produce several phytotoxins including coronatine, syringomycin, syringopeptin, phaseolotoxin, tabtoxin, and mangotoxin [25]. Quorum sensing (QS) signaling and its role in virulence has also been studied in several *Pseudomonas* species including *P. aeruginosa* [26-29], *P. syringae* pv. *syringae* [30] and *P. fuscovaginae* [31].

To our knowledge no genetic and molecular studies or screening for virulence-associated systems/functions in *Pfv* have been reported. Only a few biochemical studies have shown the production of three phytotoxins; namely syringotoxin, fuscopeptin A (FP-A) and fuscopeptin B (FP-B) [32,33] which have been shown to be able to generate the disease symptoms. We reported the role of the two QS systems in causing brown sheath rot by *Pfv* in rice [31] and have also determined the first draft genome sequence of a highly virulent *Pfv* strain [34]. In this study an *in planta* screening of 1000 genomic Tn*5* mutants has provided some insight into the virulence associated functions in *Pfv*.

## Results and discussion

### Screening of *P. fuscovaginae* Tn*5* mutants for altered virulence *in planta*

As there are no major reports regarding virulence functions of this emerging phytopathogen, we performed an *in planta* screen of 1000 Tn*5* genomic mutants to identify genes and/or pathways that might influence *Pfv* virulence potential. A Tn*5* mutant bank of *Pfv* was generated as described in the Materials and Methods section and 1000 insertion mutants randomly selected and numbered from 1 to 1000 were tested for virulence on plants of *Chenopodium quinoa*. In this screen *C. quinoa* was chosen as a plant model over rice because the infection protocol for *Pfv* is simpler to perform in *C. quinoa* compared to rice and therefore more suitable for a high-throughput screen involving many mutants [31]. Plant inoculations were performed as described in the Materials and Methods section and virulence was assessed using the virulence score from 0 to 5 as previously described [31] and presented here in Additional file 1. In the first round of screening we obtained 83 mutants that were altered for virulence compared to wild type. In order to verify the results, the 83 mutants were re-assessed for virulence in three biological replicates using two independent plants (total of six replicates). We then obtained a total of 9 mutants (Table 1) that displayed consistent and a significantly reduced virulence compared to wild type *Pfv* (Table 2). None of the mutants were affected in their growth pattern when grown in liquid rich media (data not shown).

**Table 1:** **Bacterial strains used in this study**

**Strains**	**Relevant characteristics** ^**a**^	**Reference/** **source**
*E. coli*.		
DH_5_a	Cloning strain, Nal^r^	[35]
PRK2013	Helper strain for tri-parental conjugation, Km^r^	[36]
*Pseudomonas fuscovaginae* (*Pfv*)		
*Pfv* UPB0736 (NCPPB 3871) (WT)	Wild-type strain isolated from diseased rice in Madagascar; Nf^r^, Amp^r^	[34]
*Pfv* 80	80:: Tn*5* of *Pfv* UPB0736; Nf^r^, Km^r^	This study
*Pfv* 90	90:: Tn*5* of *Pfv* UPB0736; Nf^r^, Km^r^	This study
*Pfv* 102	102:: Tn*5* of *Pfv* UPB0736; Nf^r^, Km^r^	This study
*Pfv* 169	169:: Tn*5* of *Pfv* UPB0736; Nf^r^, Km^r^	This study
*Pfv* 188	188:: Tn*5* of *Pfv* UPB0736; Nf^r^, Km^r^	This study
*Pfv* 270	270:: Tn*5* of *Pfv* UPB0736; Nf^r^, Km^r^	This study
*Pfv* 420	420:: Tn*5* of *Pfv* UPB0736; Nf^r^, Km^r^	This study
*Pfv* 445	445:: Tn*5* of *Pfv* UPB0736; Nf^r^, Km^r^	This study
*Pfv* 480	480:: Tn*5* of *Pfv* UPB0736; Nf^r^, Km^r^	This study
*Pfv* 80-pKNOCK	80:: pKNOCK mutant of *Pfv* UPB0736; Nf^r^, Km^r^	This study
*Pfv* 90-pKNOCK	90:: pKNOCK mutant of *Pfv* UPB0736; Nf^r^, Km^r^	This study
*Pfv* 102-pKNOCK	102:: pKNOCK mutant of *Pfv* UPB0736; Nf^r^, Km^r^	This study
*Pfv* 169-pKNOCK	169:: pKNOCK mutant of *Pfv* UPB0736; Nf^r^, Km^r^	This study
*Pfv* 188-pKNOCK	188:: pKNOCK mutant of *Pfv* UPB0736; Nf^r^, Km^r^	This study
*Pfv* 270-pKNOCK	270:: pKNOCK mutant of *Pfv* UPB0736; Nf^r^, Km^r^	This study
*Pfv* 420-pKNOCK	420:: pKNOCK mutant of *Pfv* UPB0736; Nf^r^, Km^r^	This study
*Pfv* 445-pKNOCK	445:: pKNOCK mutant of *Pfv* UPB0736; Nf^r^, Km^r^	This study
*Pfv* 480-pKNOCK	480:: pKNOCK mutant of *Pfv* UPB0736; Nf^r^, Km^r^	This study
*Pfv* 90 + pCos90	pCos90:: *Pfv* 90; Nf^r^, Km^r^,Tc^r^	This study
*Pfv* 420 + pCos420	pCos420:: *Pfv* 420; Nf^r^, Km^r^,Tc^r^	This study
*Pfv* 445 + pCos445	pCos445:: *Pfv* 445; Nf^r^, Km^r^,Tc^r^	This study

^a^;Nal^r^, Nf^r^ Km^r^, Tc^r^ and Amp^r^ indicates nalidixic acid, nitrofurantoin, kanamycin, tetracycline and ampicillin respectively.

**Table 2:** **Virulence screening of Tn** ***5*** **transposon mutants of** ***P. fuscovaginae*** **in** ***C. quinoa*** **plants**

***Pseudomonas fuscovaginae*** **(** ***Pfv*** **)** **strains**	**Lesion scores in screen I** **(Average ± S.D.)**	**Lesion scores in screen II** **(Average ± S.D.)**
*Pfv* UPB0736 (WT)	5, 5, 5 (5 ± 0)	5, 5, 5, 5, 5, 5 (5 ± 0)
*Pfv* 80	0, 1, 1 (0.66 ± 0.58)^a^	2, 2, 2, 2, 2, 3 (2.16 ± 0.40)^a^
*Pfv* 90	1, 1, 1 (1 ± 0)^a^	0, 0, 0, 0, 0, 1 (0.16 ± 0.40)^a^
*Pfv* 102	0, 0, 0 (0 ± 0)^a^	0, 1, 1, 2, 2, 3 (1.50 ± 1.05)^a^
*Pfv* 169	0, 0, 0 (0 ± 0)^a^	0, 0, 0, 0, 0, 0 (0 ± 0)^a^
*Pfv* 188	0, 0, 0 (0 ± 0)^a^	0, 0, 0, 0, 0, 0 (0 ± 0)^a^
*Pfv* 270	1, 1, 1 (1 ± 0)^a^	1, 1, 1, 1, 1, 1 (1 ± 0)^a^
*Pfv* 420	2, 2, 3 (2.33 ± 0.58)^a^	2, 2, 3, 3, 3, 3 (2.66 ± 0.52)^a^
*Pfv* 445	1, 1, 1 (1 ± 0)^a^	2, 2, 2, 2, 2, 2 (2 ± 0)^a^
*Pfv* 480	1, 1, 1 (1 ± 0)^a^	1, 1, 1, 1, 1, 1 (1 ± 0)^a^

Table showing disease severity for wild type and selected Tn*5* mutants of *Pfv* based on their rating score in *C. quinoa* plants. A two-tailed, paired ‘*t*’ test with 95% of confidence intervals for independent means was performed between the wild type and each of Tn*5* mutants. ^a^; significant difference to WT at *P* <0.05.

**Figure 1:**
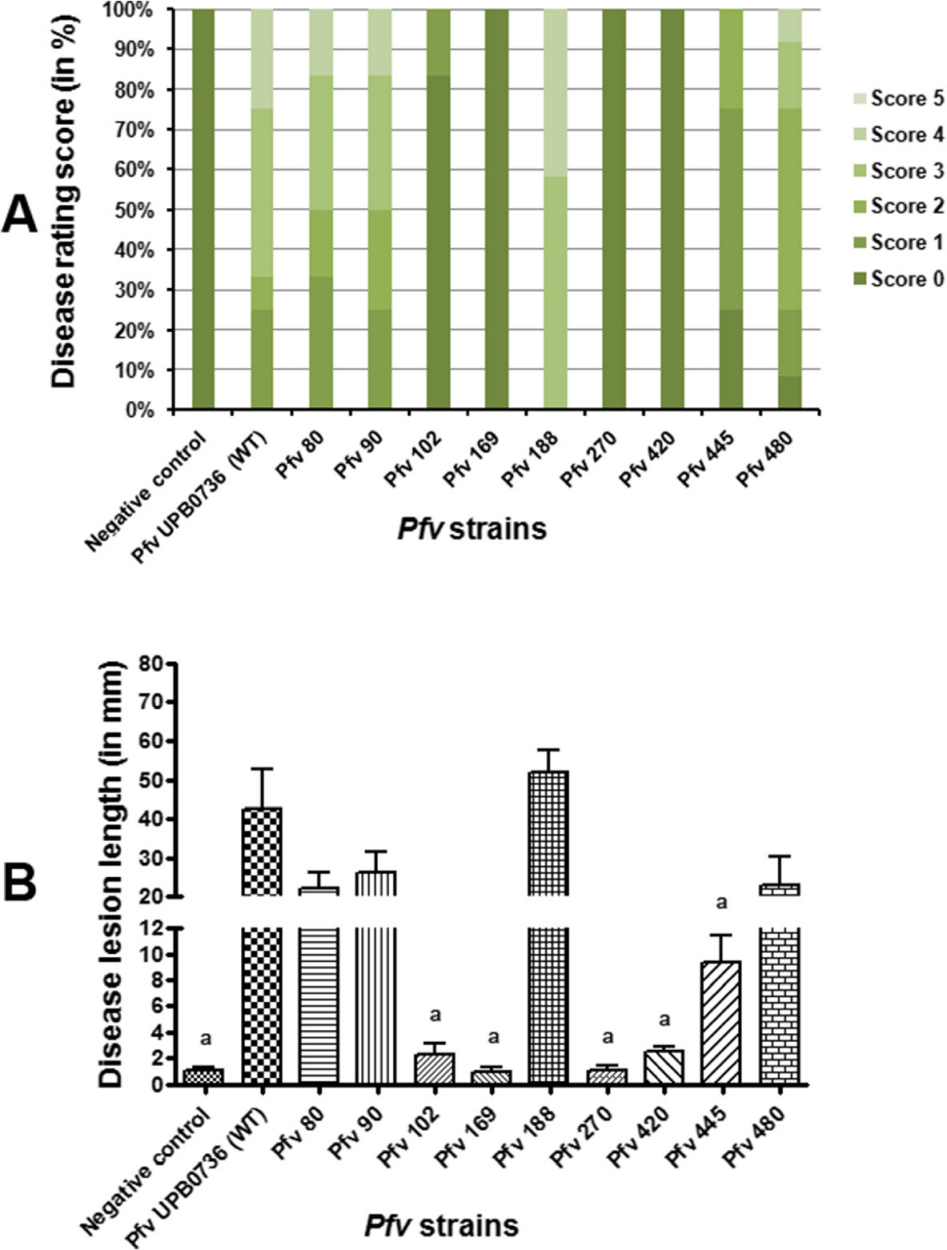
**Virulence score of selected Tn** ***5*** **mutants in rice.** Five week old susceptible rice cultivar Co-39 plantlets were inoculated using a 1 ml syringe with 100 μl of the following *Pfv* strains: *Pfv* UPB0736 (WT), *Pfv* 80, *Pfv* 90, *Pfv* 102, *Pfv* 169, *Pfv* 188, *Pfv* 270, *Pfv* 420, *Pfv* 445 and *Pfv* 480. 0.15 M saline solution was used as negative control. Data for rating score and lesion lengths were taken 8 days post inoculation. **A**. Disease severity based on rating score. Figure showing disease severity (in %) for wild type and Tn*5* mutants of *Pfv* based on their rating score from 0 to 5 in rice plants. **B**. Disease severity based on lesion length. Figure showing disease severity (lesion length in mm) for wild type and Tn*5* mutants of *Pfv* based on their lesion length score. Error bars indicate the standard deviation for readings from at least 12 inoculated leaves. Similar results were obtained in independent experiments (data not shown). A two-tailed, paired ‘*t*’ test with 95% of confidence intervals for independent means was performed between the wild type and each of Tn*5* mutants. a; significant difference to WT at *P* <0.05.

In order to verify the virulence deficiency of identified mutants, infection assays were carried out using both *C. quinoa* and rice plants. It was of interest to test all the identified mutants in both models as the strain *Pfv* UPB0736 was first isolated as a rice pathogen. Rice infection was performed by syringe inoculation (100 μl of 10^8^ cfu/ml) as described in the Materials and Methods section and virulence was assessed using the virulence score from 0 to 5 and also by measuring the lesion length (as presented in Additional file 2). In rice, 5 out of the 9 selected mutants had similar behaviour as in *C. quinoa*, being reduced in virulence when inoculated with a higher dose of bacteria (100 μl of 10^8^ cfu/ml). Three other mutants were also found with reduced virulence, although the virulence level was not significantly different compared to wild type. Surprisingly, *Pfv* 188 on the other hand showed a similar level of virulence compared to wild type in rice (Figure 1).

**Figure 2:**
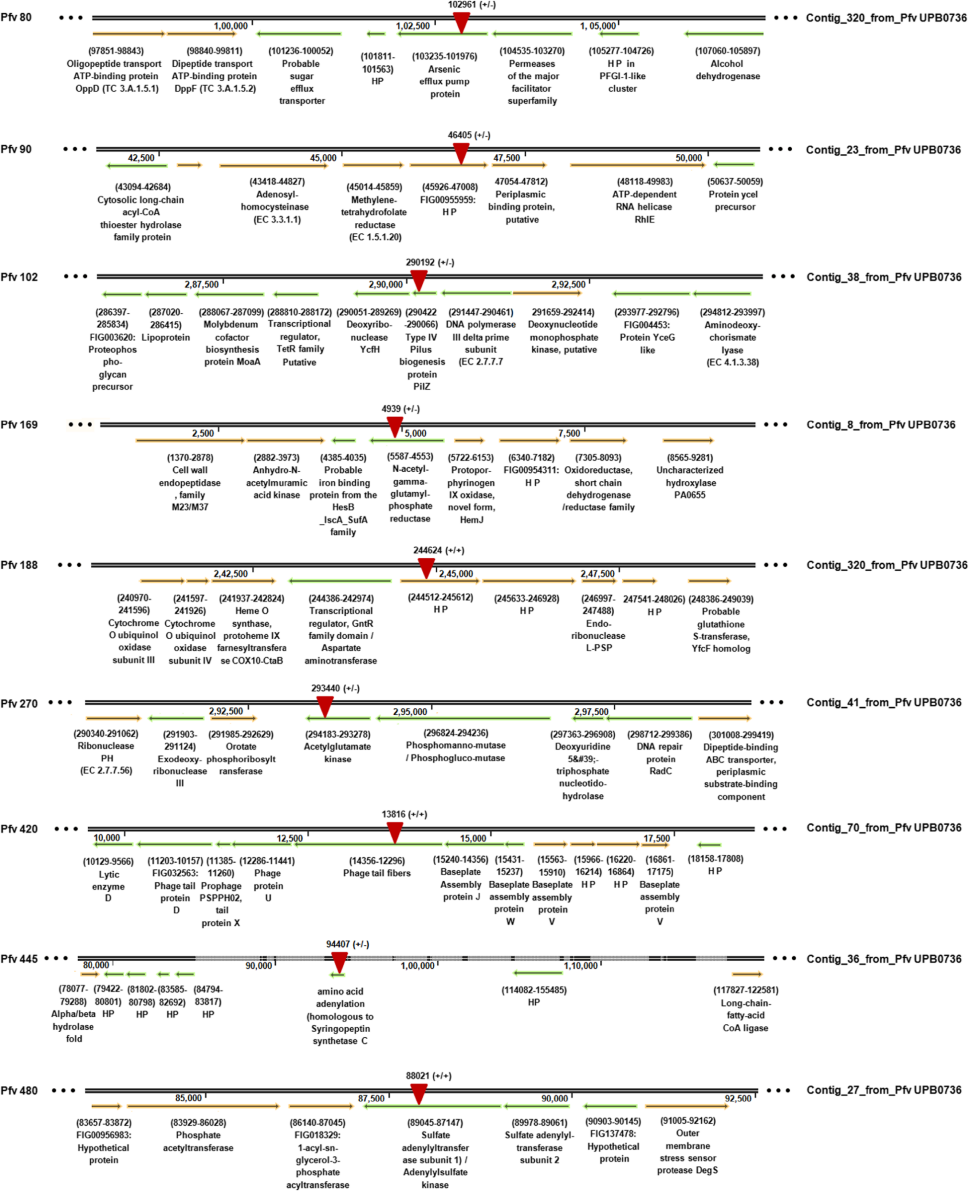
**Localization of Tn** ***5*** **insertions in identified** ***Pfv*** **UPB0736 mutants.** Localization of Tn*5* insertion for the nine mutants in *Pfv* UPB0736 draft genome. A red coloured triangle indicates the position of Tn*5* insertion. +/- sign indicates the orientation of Tn*5* insertion respective to the orientation of ORF. ORF with + or - orientation are indicated in brown and green colour respectively. Each ORF has been indicated for its size and name just below to each ORF arrow. Flanking region of Tn*5* insertion for each contig is shown by a solid line with respective positions. Stretches of dotted black line in *Pfv* 445 indicates unassigned nucleotides (n). Hypothetical proteins are abbreviated as HP.

We localized the Tn*5* insertion site in these selected nine Tn*5* mutants and mapped their insertion position in the *Pfv* UPB0736 draft genome (Figure 2). The nine Tn*5* mutants were localized in genes coding for the following features: an arsenic pump efflux (*Pfv* 80), two hypothetical proteins (*Pfv* 90; *Pfv* 188), the type IV pilus biogenesis protein, PilZ (*Pfv* 102), an N-acetyl-gamma-glutamyl-phosphate reductase (*Pfv* 169), an acetylglutamate kinase (*Pfv* 270), a phage tail fiber homolog protein (*Pfv* 420), a syringopeptin synthatase C homolog (*Pfv* 445) and a bifunctional sulphate adenylyltransferase subunit 1 (*Pfv* 480) (Figure 2).

### Validation of the genetic screening by re-generation of knock-out mutants in the same loci and their genetic complementation

**Figure 3:**
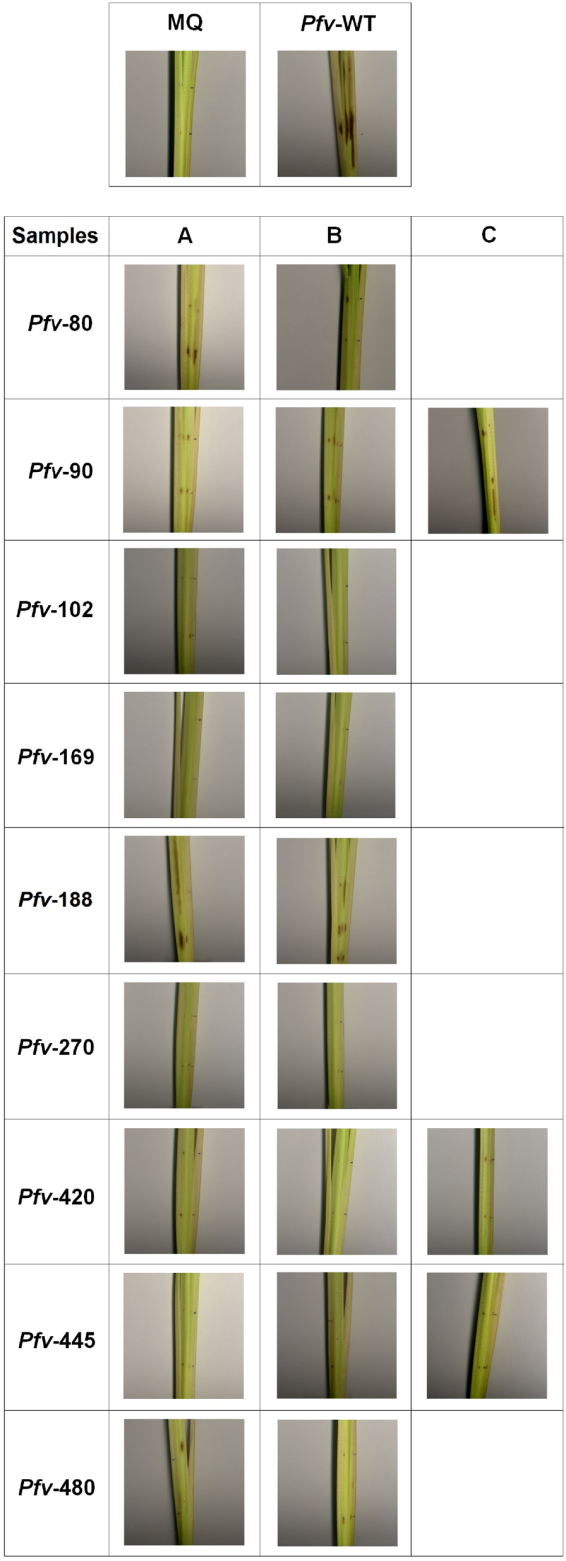
**Virulence phenotype of selected** ***Pfv*** **strains in rice.** Five week old susceptible rice cultivar Baldo were pin prick inoculated by dipping into 10^6^ cfu/ml inoculums of *Pfv* strains. **A**: Tn*5* mutants *Pfv* 80, *Pfv* 90, *Pfv* 102, *Pfv* 169, *Pfv* 188, *Pfv* 270, *Pfv* 420, *Pfv* 445 and *Pfv* 480. **B**: knock-out mutants of *Pfv* 80, *Pfv* 90, *Pfv* 102, *Pfv* 169, *Pfv* 188, *Pfv* 270, *Pfv* 420, *Pfv* 445 and *Pfv* 480. **C**: complemented strains *Pfv* 90 + pCos 90, *Pfv* 420 + pCos 420 and *Pfv* 445 + pCos 445. *Pfv* UPB0736 (WT) and MQ water were used as positive and negative control respectively. Figure showing the disease symptoms were taken 10 days post inoculation.

**Figure 4:**
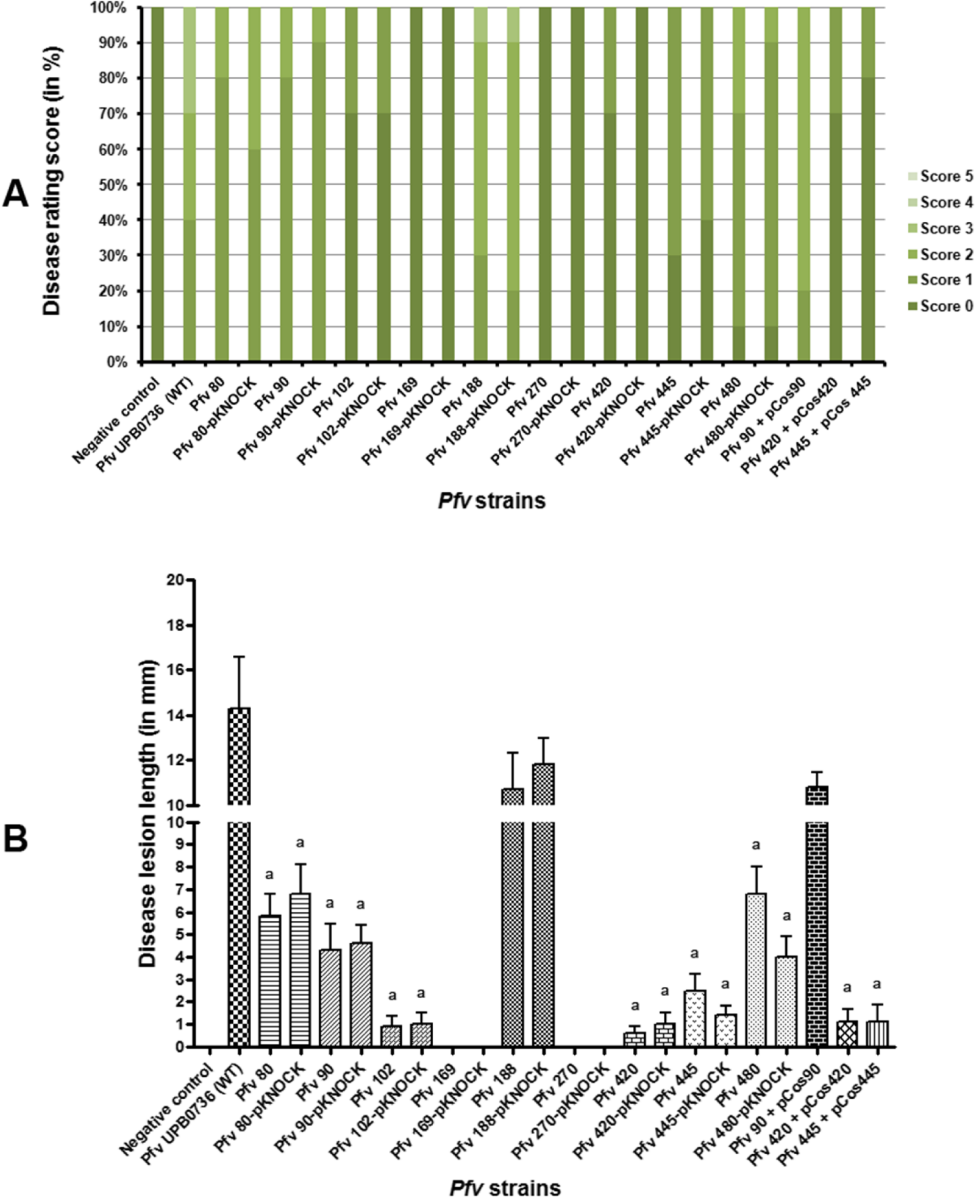
**Virulence score of selected** ***Pfv*** **strains in rice.** Five week old susceptible rice cultivar Baldo were pin prick inoculated by dipping into 10^6^ cfu/ml inoculums of following *Pfv* strains: *Pfv* UPB0736 (WT), nine Tn*5* mutants, the respective nine knock-outs and three complemented strains. Sterile MQ water was used as negative control. Data for rating score and lesion lengths were taken 10 days post inoculation. **A**. Disease severity based on rating score. Figure showing disease severity (in %) for *Pfv* strains based on their rating score from 0 to 5 in rice plants. **B**. Disease severity based on lesion length. Figure showing disease severity (lesion length in mm) for *Pfv* strains based on their lesion length score. Error bars indicate the standard deviation for readings from at least 10 inoculated leaves. Similar results were obtained in independent experiments. A two-tailed, paired ‘*t*’ test with 95% of confidence intervals for independent means was performed between the wild type and each of Tn*5* mutants. a; significant difference to WT at *P* <0.05.

In order to further verify the virulence phenotype of selected Tn*5* mutants, all mutants in the nine loci were independently re-generated via homologous recombination as described in the Materials and Methods section. In addition we also complemented three Tn*5* mutants (*Pfv* 90, *Pfv* 420 and *Pfv* 445) by identifying the genomic region harbouring each of the three loci from a cosmid library. We re-assessed the virulence phenotype of the nine Tn*5* mutants, their corresponding re-generated mutants and 3 complemented strains in rice plants by pin prick inoculation (this type of infection results in the inoculation of a lower dose of bacteria as the sterile pin was dipped in a suspension of 10^6^ cfu/ml). Eight of the nine Tn*5* mutants and their respective re-generated knock-outs showed similar results displaying virulence deficiency (P <0.05) as observed when inoculated with higher doses of bacteria in rice. Whereas *Pfv* 188 again displayed virulence symptoms similar to wild type strain as obtained with syringe inoculations (Figures 3 and 4). With respect to complementation in these experiments, the mutant *Pfv* 90, harbouring the cosmid clone carrying the corresponding wild-type locus, regained virulence completely. On the other hand the virulence assays with mutants *Pfv* 420 and 445 harbouring cosmid clones isolated from the gene bank did not result in complementation (Figures 3 and 4). Mutant *Pfv* 445 had the Tn*5* inserted in a gene homologous to the one coding for the syringopeptin C of *P. syringae* pv. *syringae* (*Pss*) B728a, thus this gene is most probably involved in the biosynthesis of one of the fuscopeptins produced by *Pfv*. Peptide synthetases are very large ORFs; for example in *Pss* B728a the three syringopeptin genes *sypA* (16119 bp), *sypB* (16410 bp) and *sypC* (40614 bp) are at least 16 kb in size (Additional file 3). The Tn*5* insertion region in mutant *Pfv* 445 was found in a gene homologous to syringopeptin C of *Pss* B728a. This homologous gene in the *Pfv* UPB0736 draft genome sequence contained stretches of unassigned nucleotides and it is likely to be an unusually large ORF. It is therefore possible that the cosmid clone used did not harbour the complete gene hence it could not complement the mutant *Pfv* 445. Mutant *Pfv* 420 had the Tn*5* transposon insertion in a gene encoding for a protein with significant homology to a phage tail fiber. In *Pfv* this gene is located in a cluster of genes with phage related functions that are probably part of an operon. Again, lack of complementation could be due to the cosmid clone not containing all the genetic material necessary for the complementation. Another possibility for not having complemented the virulence phenotype of *Pfv* 420 and *Pfv* 445 could be due to multicopy allele effects of these genes which may cause instability or fitness cost. In summary, 8/9 mutants identified using *C. quinoa* as infection model were also found affected for virulence in a similar manner in rice (except *Pfv* 188) when inoculated with low doses of bacteria. The same profile of virulence in rice was also obtained with independently generated mutants in the same loci as the identified virulence defective mutants; all these data further confirm the results of the genetic screen and indicate that the inactivated functions in the identified mutants are directly or indirectly associated with *Pfv* virulence.

### The nine genetic loci identified in the screen and their inference in virulence

Here below we describe the nine loci in which the Tn*5* insertions were located in relation to their potential role in virulence.

#### Virulence deficient mutants Pfv 80, 169, 270 and 480 have transposon insertions in genes involved in various metabolic functions

*Pfv* 80: the Tn*5* was localized in a gene that displays significant identity to an arsenic efflux pump protein (Figure 2). Arsenic is a toxic metalloid and resistance to this metal has already been described in Gram-positive and also in Gram-negative bacteria [37]. We do not know the exact mechanism of involvement in virulence for arsenic pump efflux protein in *Pfv*. However, being a toxic metal, export of arsenic through this efflux protein could be essential for a proper metabolic function and survival of the bacterial cell. It is possible that the inability of the *Pfv* mutant bacterium to expel this or a related toxic metal ion from the cytoplasm diminishes its viability *in planta* and thus makes it less virulent or less fit for growth in this environment compared to the wild type *Pfv*. Virulence studies in the grapevine pathogen, *Xylella fastidiosa* and in the fire blight pathogen, *Erwinia amylovora*, *tolC* mutant affected for efflux pump functions have shown their involvement in virulence and *in planta* fitness [38,39]. It is possible that these efflux pumps are involved in exporting heavy metals, antimicrobials or harmful plant phenolic compounds which are released as part of the plant defense response.

*Pfv* 169 and *Pfv* 270: In both mutants *Pfv* 169 and *Pfv* 270, the Tn*5* was located in genes involved in the biosynthesis of the amino acid arginine [38]. *Pfv* 169 and *Pfv* 270 were found mutated in an N-acetyl-γ-glutamyl-phosphate reductase gene (*argC*) and N-acetylglutamate kinase gene (*argB*) respectively. The two enzymes catalyze the conversion of N-acetylglutamate in N-acetylglutamate semialdehyde, via N-acetylglutamyl phosphate. Specifically, N-acetylglutamate kinase encodes the key enzyme for the biosynthesis of arginine and is inhibited by the reaction product [39,40]. In order to further verify that the two isolated mutants were affected for arginine biosynthesis, an arginine auxotrophy assay was performed as described in the Materials and Methods section. *Pfv* 169 and *Pfv* 270 mutants and their respective re-generated mutants were found affected for growth on M9 agar plates lacking the amino acid arginine. Supplementation of arginine in M9 agar restored the growth defect of *Pfv* 169 and *Pfv* 270 mutants and their respective re-generated mutants (Additional file 4). The chemical complementation by arginine supplementation further confirmed that the two mutants affect the biosynthesis are involved in arginine biosynthesis pathway. Besides having a crucial role in metabolism and growth, arginine was also shown to have a role in virulence. Arginine is one of the components of ethylene biosynthesis and together with oxoglutarate, is used by the ethylene forming enzyme (EFE) to produce ethylene. Mutants in *efe* no longer produce ethylene and were found virulence deficient in *P. syringae* pvs. *glycinea and phaseolicola* [41]. Interestingly, a homolog of *efe* is present in *Pfv* UPB0736 draft genome (data not shown). Arginine is also a fundamental part of the signal peptide that directs the protein to the transport system called twin-arginine translocation system. The consensus sequence of the proteins harbouring the double arginine motif, contains two arginine repeated Ser/Thr-Arg-Arg-X-Phe-Leu-Lys. Furthermore, mutants for the twin-arginine translocation system in *P. syringae* spp. showed reduced viability and virulence *in planta* [42,43]. It is therefore possible that the reduced virulence of mutants *Pfv* 169 and 270 is caused not only by a deficiency in the metabolism of arginine but also due to a role directly related to pathogenesis via ethylene and protein transport.

*Pfv* 480: Mutant *Pfv* 480 had a mutation in a gene that encodes a bi-functional protein with two enzymatic activities: sulfate adenylyltransferase and adenylylsulfate kinase. Both of these activities are important in the metabolism of sulfur. The sulfate adenylyltransferase catalyzes the first intracellular reaction for the assimilation of sulfur with the use of a molecule of ATP and leading to the formation of adenosine-5-phosphosulfate (APS). This compound is pivotal for the biosynthesis pathway of amino acids that contain sulfur, namely cysteine and methionine. The adenylylsulfate kinase utilized the same substrate as APS catalyzing its conversion into 3'-phosphoadenosine 5'-phosphosulfate (PAPS) using ATP molecules. PAPS is a universal donor of the sulfate group and is used as the substrate for important enzymes such as sulfotransferase [44]. It is currently unknown how these enzymes crucial for the metabolism of sulfur are involved in virulence.

#### Virulence deficient mutant Pfv 102 is affected in type IV pili biosynthesis

The Tn*5* transposon localized in the *Pfv* 102 mutant was found in Type IV pilus biogenesis gene *pilZ* encoding a protein that displayed significant identity (82 to 92%) with PilZ of other pseudomonads. The PilZ protein is one of the several Pil proteins that are associated with type IV pili biosynthesis. PilZ is the only protein that is not part of the pili biosynthesis operon and is located as a single transcriptional unit in the genome of pseudomonads. Type IV pili genes are found not only in pseudomonads but also in other Gram-negative bacteria including xanthomonads and are implicated in a wide spectrum of roles including adhesion, motility, secretion and virulence. The role of PilZ in the formation of Type IV pili has not yet been well established. In some cases, knock-out mutants are incapable of secreting a protein which constitutes the pilus whereas in other cases PilZ does not seem to be essential for the formation of the pilus and for bacterial movement [45-47]. In recent years, sequencing of several bacterial genomes has revealed the presence of a PilZ domain in many proteins and has associated the function of this domain with the binding of the second-messenger cyclic guanosine monophosphate [46]. The c-di-GMP regulates many functions including aggregation, biofilm formation, EPS production, adhesion, movement and virulence [48]. It is possible that PilZ in *Pfv* can influence the signalling cascade mediated by c-di-GMP that could be involved in the pathogenic process.

#### Virulence deficient mutant Pfv 420 is most likely affected in type VI secretion machinery

Tn*5* transposon localized in *Pfv* 420 mutant was found in a gene encoding a protein annotated as a phage tail fiber in *Pfv* UPB0736. This gene was found adjacent to other loci encoding phage related proteins; namely the phage protein U and baseplate assembly protein J (Figure 2). Phage related functions are reported to be present in 25% of the Gram-negative bacterial genomes [49]. These genes encode for protein components that are structurally similar to tailed bacteriophages and are possibly involved in synthesizing a specialized contractile injection system that is known as Type VI secretion system (T6SS). Based on structural similarities, T6SS appeared as an inverted phage tail on the surface of the bacterial cell and it secretes effector proteins into the extracellular milieu or injects them directly into host cells by a puncturing mechanism [50-53]. A common evolutionary history has been proposed for the two injection machines present in bacteria and bacteriophages [52,54]. In plant associated bacteria, T6SS has been implicated in several functions including tumorigenesis in *Agrobacterium* [55], programmed cell death in the filamentous fungus *Neurospora crassa* by *Pss* B728a [56] and virulence in *Pectobacterium* [57]. Interestingly, *P. fuscovaginae* UPB0736 possesses a T6SS (data not shown). Consequently the Tn*5* mutation in phage tail fiber-like protein most likely results in a non-functional T6SS secretion machinery for delivery of effector proteins in *Pfv* UPB0736 thus affecting virulence.

#### Virulence deficient mutant Pfv 445 is most likely affected in phytotoxin production

In this mutant the sequence of the gene inactivated by Tn*5* displays 96% identity with the gene of *P. syringae* that encodes an enzyme called syringopeptin synthetase C (*sypC*). In *P. syringae* this gene is 40614 bp long and is part of a gene cluster of 73800 bp that also includes syringopeptin synthetase A (*sypA*) and syringopeptin synthetase B (*sypB*) [58] (Additional file 3). The genetic organization of these loci reveals that several genes flanking this locus are conserved among other *Pseudomonas* cyclic lipopeptides (CLP) biosynthesis clusters, including two genes encoding a putative macrolide transporter MacA and MacB. Genes encoding MacA and MacB have been reported in syringopeptin, syringomycin [59,60] and entolycin biosynthesis gene clusters [61] and they were also found in *Pfv* UPB0736 (data not shown). The three peptide synthetases are responsible for the biosynthesis of non-ribosomal syringopeptin, which represents one of the major virulence factors in *P. syringae* [62]. *Pfv* produces phytotoxins which are similar to syringopeptin and are called fuscopeptins A and B [63]. These lipodepsipeptides show numerous structural and functional characteristics common to syringopeptin isolated from *P. syringae*. The distinguishing feature of the mechanism of action of these lipodepsipeptides is their ability to interact with biological membranes. The amphipathic nature of these molecules allows their insertion into the lipid bilayer, with the consequent formation of ion channels that cause the alteration of the membrane potential and in turn the loss of intracellular material [64]. The leakage from the host cells enriches the intercellular fluid with sucrose, amino acids, inorganic ions and other supplements that could be supporting the bacterial multiplication [63]. These phytotoxins are also able to play a role on disease symptoms by inducing injuries in the host plant. Syringotoxin has also properties of surfactant, fungicidal action and alteration in proton gradient. The antagonistic activity is likely to increase the competitive ability of *Pfv* against other colonizers of leaf surfaces. Although in earlier studies three *Pfv* phytotoxins were characterized biochemically, here we report that a mutant in a vital gene for the biosynthesis of at least one of them results in *Pfv* being significantly less virulent.

#### Virulence deficient mutant affected in hypothetical proteins (Pfv 90 and Pfv 188)

*Pfv* 90 mutant had a Tn*5* insertion localised in a gene encoding for a hypothetical protein; adjacent to this ORF there is a methylene tetrahydrofolate reductase gene and a gene encoding a periplasmic binding protein (Figure 2). The spacing between the gene encoding the hypothetical protein and the next ORF (periplasmic binding protein) is only 46 bp thus we cannot exclude that the *Pfv* 90 mutant phenotype that could be associated with periplasmic binding gene located downstream in an operonic organization. Mutant *Pfv* 188 on the other hand, had the Tn*5* insertion localized in a gene coding for a hypothetical protein that is flanked by a gene coding for a transcriptional regulator possessing a GntR family domain on one side and by a gene encoding a hypothetical protein on the other side. The gene encoding the neighbouring hypothetical protein is 21 bp away from the gene in which Tn*5* insertion is localised hence they could be organized in operonic structure. Complementation using cosmid clones resulted in the restoration of virulence for *Pfv* 90 indicating that *Pfv* 90 locus was responsible for causing a virulence deficiency. We did not find any information related to virulence functions for *Pfv* 90 in the literature suggesting that this is a novel gene and is implicated in virulence in *Pfv*.

## Conclusions

Despite the importance of *Pfv* as an emerging pathogen worldwide, to date no major studies have been performed to understand the mechanisms of *Pfv* pathogenicity. In 2012 we reported the first genome sequence of a highly virulent strain UPB0736 [34] and since then the genome sequence of another strain has been published [65] and many more genomes will most probably be sequenced in the future. In this study, we sought to identify and characterize some of the genes involved in *Pfv* virulence through an *in planta* screening of 1000 Tn*5* mutants; nine mutants that showed virulence deficiency compared to the wild type were identified. The inactivated loci in these mutants include some metabolic functions and also some known virulence associated functions such as type IV pilus biogenesis protein PilZ, T6SS machinery and syringopeptin synthetase. The results of this study highlight the fact that *Pfv* might share features of some of its virulence mechanisms with other phytopathogens. In addition, new loci never reported as being involved in virulence and encoding for hypothetical proteins have been found. Genome mining with future virulence studies will further highlight the mechanisms of virulence of this broad host range emerging phytopathogen.

## Methods

### Bacterial strains, plasmids, and culture media

The bacterial strains used in this work are listed in Table 1. The plasmids used and generated in this study are listed in Additional file 5. *Pseudomonas fuscovaginae* (*Pfv*) strains were grown at 28°C in Luria Bertani (LB)/King’s B (KB) medium, and *E. coli* strains were grown at 37°C in LB medium, as described previously [31]. The concentrations of antibiotics used in this study were as follows: Nitrofurantoin (Nf); 100-150 μg/ml, Ampicillin (Amp); 100 μg/ml, Kanamycin (Km); 50 μg/ml, Tetracyclin (Tc) 30 μg/ml for *Pfv* and Amp; 75 μg/ml, Km; 50 μg/ml, Tc; 15 μg/ml for *E. coli*.

### Recombinant DNA techniques

Routine DNA manipulation steps such as digestion with restriction enzymes, agarose gel electrophoresis, purification of DNA fragments, ligations with T4 ligase, radioactive labelling by random priming and transformation of *E. coli* etc. were performed as described previously [66]. Colony hybridizations were performed using N + Hybond membrane (Amersham Biosciences); plasmids were purified using the EuroGold plasmid columns (Euro Clone) or with the alkaline lysis method [67]; total DNA from *Pfv* strains were isolated by Sarkosyl/Pronase lysis as described previously [68]. PCR amplifications were performed using Go-Taq DNA polymerase or pfu DNA polymerase (Promega). The oligonucleotide primers used in this study are listed in Additional file 6. Automated sequencing was performed by Macrogen sequence service (Europe). Triparental matings between *E. coli* and *Pfv* were carried out with the helper strain *E. coli* DH5α (pRK2013) [36].

### Generation of Tn*5* mutant library of *Pfv* UPB0736

Tn*5* mutagenesis was performed by using triparental matings between donor *E. coli* (pSUP2021) containing the transposon Tn*5* (Km resistance), a helper *E. coli* strain (pRK2013) and recipient *Pfv* UPB0736 strain. Briefly, *Pfv* UPB0736, donor and helper *E. coli* strains were grown overnight in 20 ml of LB media supplemented with appropriate antibiotics. Cells were pelleted, washed and re-suspended in 10 ml of sterile LB media. Absorbance of all three strains were measured and cells were mixed in the following ratio: recipient *Pfv* UPB0736, 2×10^8^ colony forming units (cfu/ml); helper *E. coli*, 4×10^9^ cfu/ml; donor *E. coli*, 4×10^9^ cfu/ml. The mixture of cells were pelleted out, re-suspended in small volume of LB media and spotted onto Hybond N Plus nylon membrane (Amersham Pharmacia Biotech) that was overlaid on LB agar. Overnight incubated cells grown at 28°C were scraped from the membrane and re-suspended in 1 mL of sterile LB media. The cell suspension (50 μl each) was plated on LB agar plates containing Nf and Km. The plates were incubated at 28°C for 2-3 days to allow the growth of transconjugants (Tn*5* mutants). The *Pfv* Tn*5* mutants were then patched onto LB agar plates with Nf and Km, grown in liquid media and a glycerol stock was prepared and stored at -80°C.

### Screening of *Pfv* UPB0736 Tn*5* mutants for virulence deficiency in *Chenopodium quinoa* (*C. quinoa*) and rice plants

One thousand independent Tn*5* mutants of *Pfv* UPB0736 were grown on fresh LB agar plates and inoculated individually in greenhouse-grown 2 week old *C. quinoa* plants. Inoculation was performed using a needle (size; 21 G × 1 ½”) by touching the strains on plates and pricking onto twigs of *C. quinoa* plants. After 5 days of inoculations, brown sheath rot disease symptoms were observed and scored on a scale of 0 to 5 as previously reported [31] and shown again here in Additional file 1. *Pfv* Tn*5* mutants with deficiency in virulence compared to wild type were subjected to a second round of screening using two independent plants.

In order to verify the virulence phenotype of selected Tn*5* mutants from the *C. quinoa* screen, mutants were re-inoculated on rice plants which is a real host for this bacterium. Rice plants (cultivar Co-39) were grown in the growth chamber at 28 ± 4°C and approximately 70% relative humidity. Along with wild type *Pfv* UPB0736, the selected Tn*5* mutants were grown for 48 hrs in KB medium. Bacterial cultures were diluted to 10^8^ cfu/ml using 0.15 M saline solution and inoculated onto five week-old rice plants using a 1 ml syringe. Inoculation was performed by injecting 100 μl of bacterial culture in the rice plantlet at 5 cm above ground. After inoculation, plantlets were kept in a humid chamber at 20-30°C for disease development. Each bacterial strain was inoculated in rice plantlets in at least 12 replicates. Eight days after inoculation, data for disease severity in lesion lengths (mm) and disease rating score (on a scale from 0 to 5) were collected and analysed for statistical significance using two-tailed, paired ‘*t*’ test with 95% of confidence intervals.

### Localization of Tn*5* insertion

Genomic DNA was isolated from selected *Pfv* Tn*5* mutants and double digested either with BamHI + EcoRI, BamHI + SacI or BamHI + ClaI. These double digested products were ligated in pBluescript (double digested with the corresponding set of enzymes), transformed into DH5a *E. coli* cells and selected on LB agar plates with ampicillin (75 μg/ml) + kanamycin (50 μg/ml). These pBluescript clones having the insertion of Tn*5* flanking regions were sequenced using Tn*5* specific Tn*5*-Ext primers (Additional file 6). Sequences obtained were subjected to homology searches using NCBI Blast and also with the draft genome sequence of *Pfv* UPB0736 using a local blast algorithm. The exact positions of Tn*5* insertion were mapped in the *Pfv* UPB0736 draft genome. We also performed arbitrary PCR using pairs of primers mentioned in Additional file 6 as previously described [69]. Arbitrary PCR products cloned in pGEM-T easy were also sequenced using pGEM-T easy vector specific primers.

### Confirmation of Tn*5* mutations

The selected Tn*5* mutants were reconstructed by deletion of the wild-type genes via homologous recombination with the use of the pKNOCK-Km suicide delivery system. Briefly, an internal fragment of each Tn*5* insertion locus was amplified by PCR using primers listed in Additional file 6 and cloned in pGEMT-easy vector. EcoRI digested internal fragments were ligated to EcoRI digested pKNOCK-Km and transformed into C118 cells yielding pKNOCK plasmids having internal fragments from selected Tn*5* loci. These pKNOCK plasmids were further used as a suicide delivery system and the nine Tn*5* mutants from *Pfv* 80 to *Pfv* 480 mutants were regenerated in the wild-type as previously described [70]. *Pfv* mutant strains were verified by PCR analysis and sequencing.

In order to complement *Pfv* UPB0736 Tn*5* mutants, a cosmid library was constructed from the *Pfv* UPB0736 strain by using the cosmid pLAFR3 [71] as a vector. Insert DNA was prepared by partial EcoRI digestion of the genomic DNA and then ligated into the corresponding site in pLAFR3. The ligated DNA was then packaged into λ phage heads using Gigapack III Gold packaging extract (Stratagene) and the phage particles were transduced to *E. coli* HB101 as recommended by the supplier. In order to identify the cosmids containing the genes of interest (90, 420 and 445), the cosmid library was screened using radio-labelled probes for Tn*5* insert regions from 90, 420 and 445 in colony hybridization. We obtained respective cosmid clones pCos 90, pCos 420 and pCos 445 in this screen. It is not known whether the cosmids contain the full length genes for 90, 420 and 445 (Additional file 5) and they were introduced in *Pfv* 90, *Pfv* 420 and *Pfv* 445 Tn*5* mutants respectively by conjugation. Positive complemented clones were selected on LBA plates supplemented with Nf, Km and Tc.

### Arginine auxotrophy assay

Stock solution of arginine-HCl (Sigma Chemicals) was prepared at a concentration of 100 mg/ml in sterile MQ water and filter sterilized. M9 agar plates with 0.2% glucose and with and without arginine (100 μg/ml) were prepared. The *Pfv* wild type strain, the two arginine biosynthesis defective Tn*5* mutants *Pfv* 169 and *Pfv* 270, their respective re-created knock-out mutants and one other Tn*5* mutant *Pfv* 102 as a control were streaked onto each of these plates and incubated at 30°C for 24-48 hrs.

### Virulence assay in rice by pin prick inoculation

*Pfv in planta* virulence assay was performed on rice plants by pin pricking method with some modifications as described previously [31]. Briefly, the seeds of a susceptible rice variety (Baldo) were surface sterilized using washes with hypochloride and sterile MQ water. Surface sterilized rice seeds were germinated on a sterile filter paper in a petriplate in the dark at 30°C. Rice seedlings of 5 to 6 cm growth stages were planted in 50 ml falcon tubes containing Hoaglands solutions with 0.5% agar. The transplanted rice plants were maintained in a climate controlled room with conditions set to 70% humidity, 16/8 hours light/dark and temperature at 28°C and watered regularly. For inoculation, *Pfv* strains were grown for 24 hrs on LBA medium supplemented with appropriate antibiotics, at 30°C. Bacterial cultures were pelleted down washed with sterile MQ water and adjusted to approximately 10^6^ cfu/ml in sterile MQ water. Four to five week old rice plants were pin prick inoculated using a sterile board pin by dipping in the bacterial inoculum. For each strain, 10 plants were inoculated in two different sites each and control plants were inoculated in the similar manner using sterile MQ water. In order to determine *in planta* virulence of *Pfv* strains, disease severity was assessed on the 10^th^ day post inoculation by measuring the browning lesion length neighboring to the zone of inoculation and also by assessing their disease rating score (scale of 0 to 5). Virulence score with average and standard deviations are presented. The statistical significance was performed using two-tailed, paired ‘*t*’ test with 95% of confidence intervals.

## Additional files

Additional file 1:
**Virulence score of** ***Pfv*** **strains in** ***Chenopodium quinoa.*** Severity scale used to evaluate disease caused by *Pfv* infection on *Chenopodium quinoa*: severity score 0; No symptoms, severity score 1; Necrosis on less than 2 mm around the puncture, severity score 2; Necrosis on 2 to 10 mm around the puncture, severity score 3; Necrosis on 2 to 10 mm around the puncture and bending of petiole, severity score 4; Collapse of the petiole and severity score 5; Wilting of the leaf. Additional file 2:
**Virulence score of** ***Pfv*** **strains in rice.** Illustration of the rating scores used in evaluating the severity of sheath rot lesions on rice inoculated with bacterial strains. A: severity score 0; No symptoms only the sign of the injection puncture, B: severity score 1; Necrosis around the puncture till 1 cm, C: severity score 2; Necrosis around the puncture and chlorosis from 1 to 2 cm, D: severity score 3; Necrosis around the puncture from 2 to 3 cm, E: severity score 4; Necrosis around the puncture from 3 to 5 cm and F: severity score 5; Necrosis around the puncture from 5 cm and above. Additional file 3:
**Syringopeptine operon in** ***Pss*** **B728a.**
Additional file 4:
**Arginine auxotrophy.** The wild type, two arginine biosynthesis defective Tn*5* mutants *Pfv* 169 and *Pfv* 270, their respective knock-out mutants and one other Tn*5* mutant *Pfv* 102 as a control were streaked onto A: LB agar plate, B: M9 plate with 2% CAS amino acid, C: M9 plate with 2% glucose and D: M9 plate with 2% glucose and 25 μg/ml of arginine-HCl. (+) and (-) indicates growth and no growth respectively. Additional file 5:
**List of plasmids used in this study.**
Additional file 6:
**List of primers used in this study.**
