# Supplementary figures and images for: Identification of virulence associated loci in the emerging broad host range plant pathogen Pseudomonas fuscovaginae
Source: BMC Microbiol. 2014 Nov 14;14:274. doi: 10.1186/s12866-014-0274-7 (PMC4237756; doi:10.1186/s12866-014-0274-7)

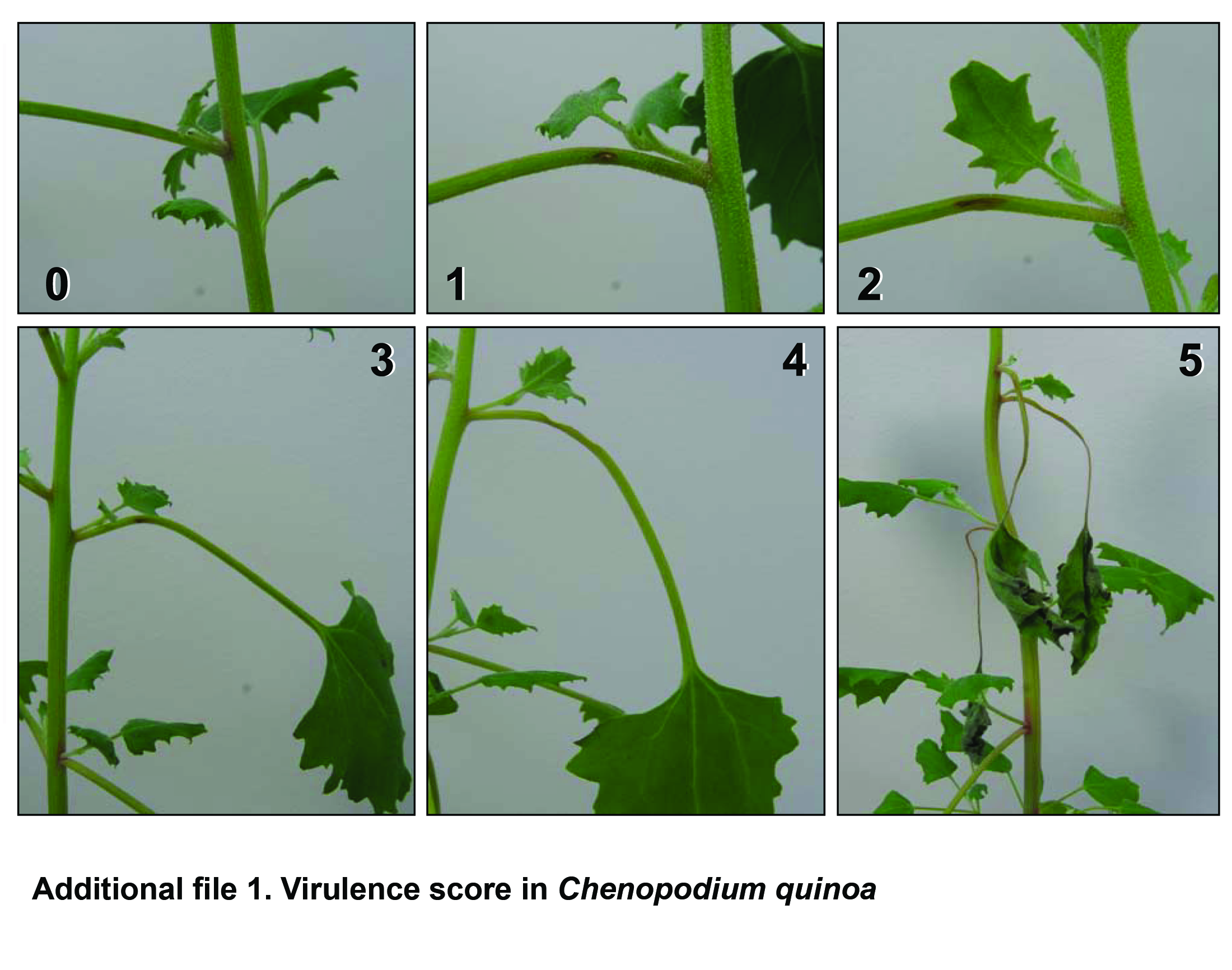

Supplement: Additional file 1: — Virulence score of Pfv strains in Chenopodium quinoa. Severity scale used to evaluate disease caused by Pfv infection on Chenopodium quinoa: severity score 0; No symptoms, severity score 1; Necrosis on less than 2 mm around the puncture, severity score 2; Necrosis on 2 to 10 mm around the puncture, severity score 3; Necrosis on 2 to 10 mm around the puncture and bending of petiole, severity score 4; Collapse of the petiole and severity score 5; Wilting of the leaf. [file 12866_2014_274_MOESM1_ESM.tiff]

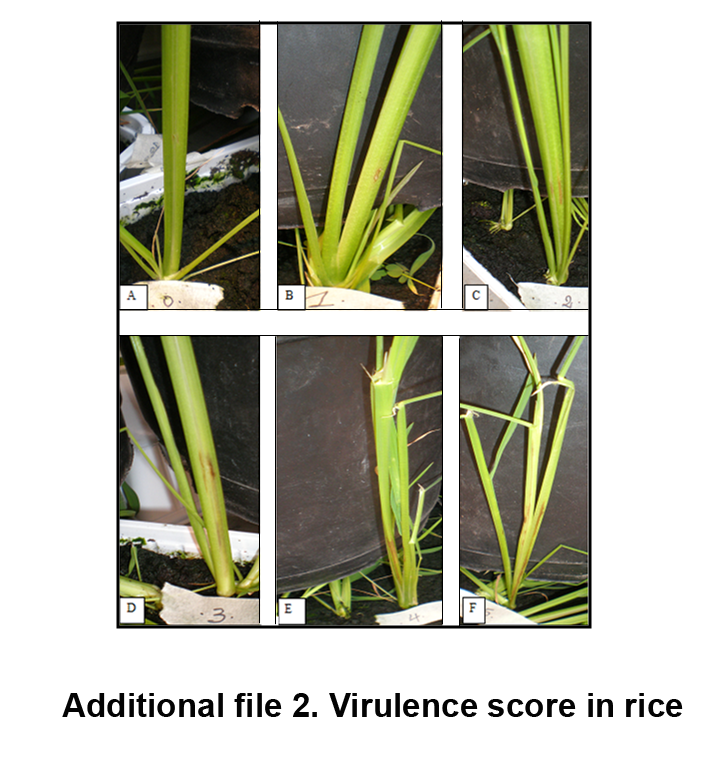

Supplement: Additional file 2: — Virulence score of Pfv strains in rice. Illustration of the rating scores used in evaluating the severity of sheath rot lesions on rice inoculated with bacterial strains. A: severity score 0; No symptoms only the sign of the injection puncture, B: severity score 1; Necrosis around the puncture till 1 cm, C: severity score 2; Necrosis around the puncture and chlorosis from 1 to 2 cm, D: severity score 3; Necrosis around the puncture from 2 to 3 cm, E: severity score 4; Necrosis around the puncture from 3 to 5 cm and F: severity score 5; Necrosis around the puncture from 5 cm and above. [file 12866_2014_274_MOESM2_ESM.tiff]

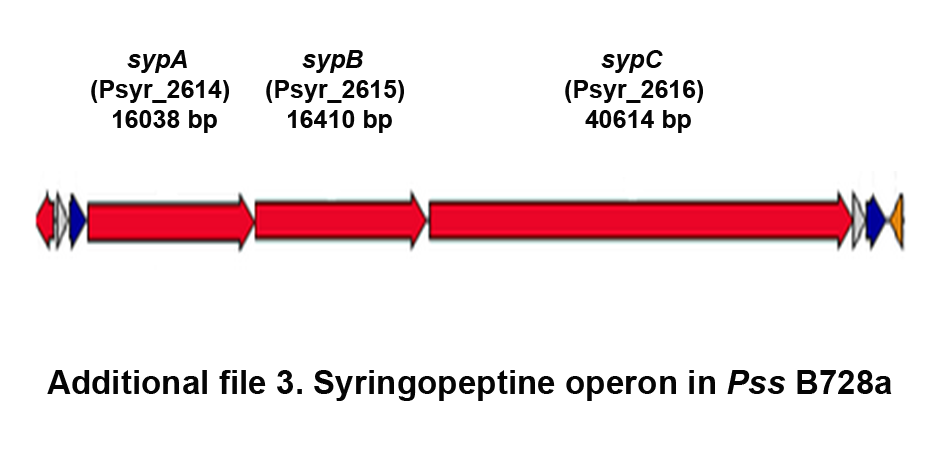

Supplement: Additional file 3: — Syringopeptine operon in Pss B728a. [file 12866_2014_274_MOESM3_ESM.tiff]

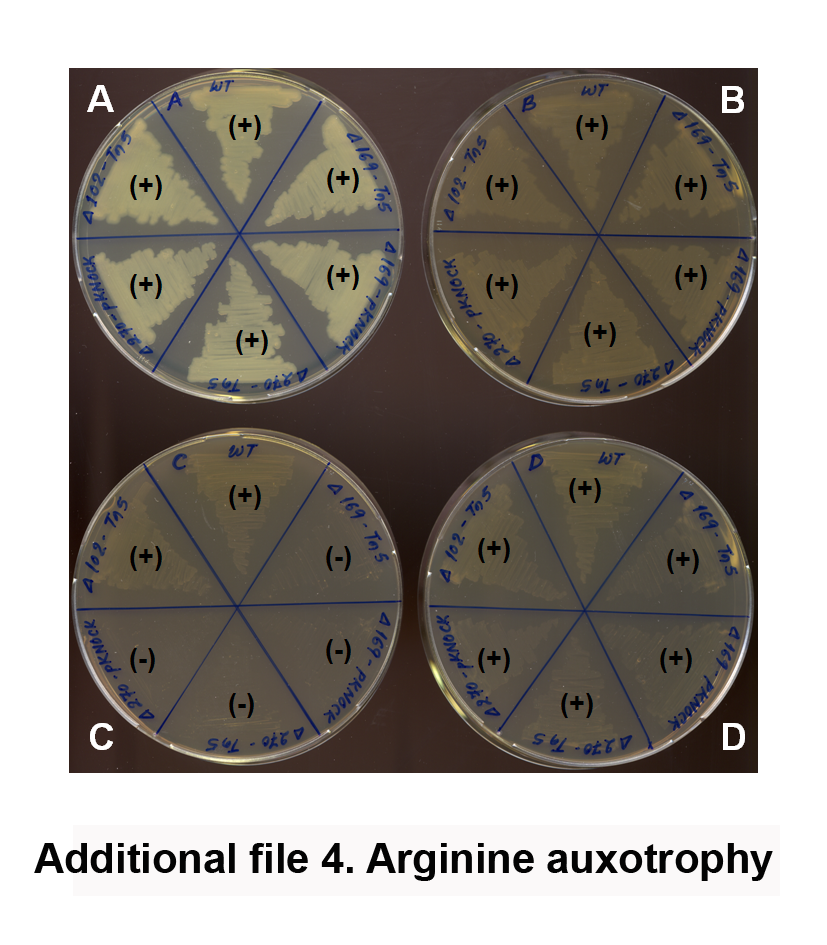

Supplement: Additional file 4: — Arginine auxotrophy. The wild type, two arginine biosynthesis defective Tn5 mutants Pfv 169 and Pfv 270, their respective knock-out mutants and one other Tn5 mutant Pfv 102 as a control were streaked onto A: LB agar plate, B: M9 plate with 2% CAS amino acid, C: M9 plate with 2% glucose and D: M9 plate with 2% glucose and 25 μg/ml of arginine-HCl. (+) and (-) indicates growth and no growth respectively. [file 12866_2014_274_MOESM4_ESM.tiff]
