# Supplementary material for: Identification of virulence associated loci in the emerging broad host range plant pathogen Pseudomonas fuscovaginae
Source: BMC Microbiol. 2014 Nov 14;14:274. doi: 10.1186/s12866-014-0274-7 (PMC4237756; doi:10.1186/s12866-014-0274-7)
Supplement: Additional file 5: — List of plasmids used in this study. [file 12866_2014_274_MOESM5_ESM.doc]

**Additional file 5. List of plasmids used in this study**

| ***Plasmids*** |  |  |
| --- | --- | --- |
| pGEMT-easy | Cloning vector; Amp^r^ | Promega |
| pBluescript | Cloning vector; Amp^r^ | Stratagene |
| pKNOCK-Km | Conjugative suicide vector; Km^r^ |  |
| pLAFR3 | Broad-host-range cloning vector, IncP1; Tc^r^ |  |
| pBS80 | pBluescript containing Tn*5* insertion region from *Pfv* 80; Amp^r^ | This study |
| pBS90 | pBluescript containing Tn*5* insertion region from *Pfv* 90; Amp^r^ | This study |
| pBS102 | pBluescript containing Tn*5* insertion region from *Pfv* 102; Amp^r^ | This study |
| pBS169 | pBluescript containing Tn*5* insertion region from *Pfv* 169; Amp^r^ | This study |
| pBS188 | pBluescript containing Tn*5* insertion region from *Pfv* 188; Amp^r^ | This study |
| pBS270 | pBluescript containing Tn*5* insertion region from *Pfv* 270; Amp^r^ | This study |
| pBS420 | pBluescript containing Tn*5* insertion region from *Pfv* 420; Amp^r^ | This study |
| pBS445 | pBluescript containing Tn*5* insertion region from *Pfv* 445; Amp^r^ | This study |
| pBS480 | pBluescript containing Tn*5* insertion region from *Pfv* 480; Amp^r^ | This study |
| pCos90 | Cosmid clone for *Pfv* 90; Tc^r^ | This study |
| pCos420 | Cosmid clone for *Pfv* 420; Tc^r^ | This study |
| pCos445 | Cosmid clone for *Pfv* 445; Tc^r^ | This study |
| pGEM80 | pGEMT-easy vector containing internal region from *Pfv* 80; Amp^r^ | This study |
| pGEM90 | pGEMT-easy vector containing internal region from *Pfv* 90; Amp^r^ | This study |
| pGEM102 | pGEMT-easy vector containing internal region from *Pfv* 102; Amp^r^ | This study |
| pGEM169 | pGEMT-easy vector containing internal region from *Pfv* 169; Amp^r^ | This study |
| pGEM188 | pGEMT-easy vector containing internal region from *Pfv* 188; Amp^r^ | This study |
| pGEM270 | pGEMT-easy vector containing internal region from *Pfv* 270; Amp^r^ | This study |
| pGEM420 | pGEMT-easy vector containing internal region from *Pfv* 420; Amp^r^ | This study |
| pGEM445 | pGEMT-easy vector containing internal region from *Pfv* 445; Amp^r^ | This study |
| pGEM480 | pGEMT-easy vector containing internal region from *Pfv* 480; Amp^r^ | This study |
| pKNOCK80 | pKNOCK-Km vector containing internal region from *Pfv* 80; Km^r^ | This study |
| pKNOCK 90 | pKNOCK-Km vector containing internal region from *Pfv* 90; Km^r^ | This study |
| pKNOCK 102 | pKNOCK-Km vector containing internal region from *Pfv* 102; Km^r^ | This study |
| pKNOCK 169 | pKNOCK-Km vector containing internal region from *Pfv* 169; Km^r^ | This study |
| pKNOCK 188 | pKNOCK-Km vector containing internal region from *Pfv* 188; Km^r^ | This study |
| pKNOCK 270 | pKNOCK-Km vector containing internal region from *Pfv* 270; Km^r^ | This study |
| pKNOCK 420 | pKNOCK-Km vector containing internal region from *Pfv* 420; Km^r^ | This study |
| pKNOCK 445 | pKNOCK-Km vector containing internal region from *Pfv* 445; Km^r^ | This study |
| pKNOCK 480 | pKNOCK-Km vector containing internal region from *Pfv* 480; Km^r^ | This study |

**^a^; Km^r^, Tc^r^ and Amp^r^ indicates for kanamycin, tetracycline and ampicillin resistance respectively.**

REFERENCES

1. Alexeyev MF: The pKNOCK series of broad-host-range mobilizable suicide vectors for gene knockout and targeted DNA insertion into the chromosome of gram-negative bacteria. *BioTechniques* 1999, 26(5):824-826, 828.

2. Staskawicz B, Dahlbeck D, Keen N, Napoli C: Molecular characterization of cloned avirulence genes from race 0 and race 1 of Pseudomonas syringae pv. glycinea. *Journal of bacteriology* 1987, 169(12):5789-5794.
