# Supplementary material for: Identification of virulence associated loci in the emerging broad host range plant pathogen Pseudomonas fuscovaginae
Source: BMC Microbiol. 2014 Nov 14;14:274. doi: 10.1186/s12866-014-0274-7 (PMC4237756; doi:10.1186/s12866-014-0274-7)
Supplement: Additional file 6: — List of primers used in this study. [file 12866_2014_274_MOESM6_ESM.doc]

**Additional file 6. List of primers used in this study**

| ***Primers*** | **Sequence (5’ to 3’)** | **Source** |
| --- | --- | --- |
| Tn5-Ext | GAACGTTACCATGTTAGGAGGTC | Lab collection |
| Tn5-Int | CGGGAAAGGTTCCGTTCAGGACGC | Lab collection |
| Arb-1 | GGCCACGCGTCGACTAGTACNNNNNNNNNNGATAT | Lab collection |
| Arb-2 | GGCCACGCGTCGACTAGTAC | Lab collection |
| SP6 | ATTTAGGTGACACTATAG | Lab collection |
| T7 | TAATACGACTCACTATAGGG | Lab collection |
| pKNOCK-New F | CTTAACCGCTGACATGGGAA | Lab collection |
| pKNOCK-New R | TTTATTCGGACACGCGTCCT | Lab collection |
| Pfv 80-Int-Fw | TTGAGCACCGGCATGGGATTA | This study |
| Pfv 80-Int-Rv | TTCGACTGGCTGGCCATGTA | This study |
| Pfv 90-Int-Fw | GGCAATCGACATCCAGGTT | This study |
| Pfv 90-Int-Rv | TTGAGCGAAAGGGAAGAGC | This study |
| Pfv 102-Int-Fw | TCGCTGACCATCAAAGACAAGTCG | This study |
| Pfv 102-Int-Rv | GCAGTATTGTCGCCGTCATTGAAC | This study |
| Pfv 169-Int-Fw | ACTCTGGGTGCCTGTGATGT | This study |
| Pfv 169-Int-Rv | GGCGAATTTCCGGCAGATGA | This study |
| Pfv 188-Int-Fw | AAGGACCAGGGGATTGTCATGT | This study |
| Pfv 188-Int-Rv | TGAGGATCAGCATCAGGCCATA | This study |
| Pfv 270-Int-Fw | TCCATCGAGAGCCGTTTCGT | This study |
| Pfv 270-Int-Rv | ACCTTGCCTTCCTTGTCCATCA | This study |
| Pfv 420-Int-Fw | TGGCCCTGGCAATGAAGTATGT | This study |
| Pfv 420-Int-Rv | CGGCAAATCATCGTCACCAGTT | This study |
| Pfv 445-Int-Fw | GTTCGGCCGGATAAGCAGGAT | This study |
| Pfv 445-Int-Rv | TCGGTGACATGGCCTTGCAAA | This study |
| Pfv 480-Int-Fw | TGAAGCCTTCGAGCATGCAC | This study |
| Pfv 480-Int-Rv | TGCCAATCTCGTTCAGTTGCAG | This study |
